# Data-Driven Prediction of Freezing of Gait Events From Stepping Data

**DOI:** 10.3389/fmedt.2020.581264

**Published:** 2020-11-20

**Authors:** Midhun Parakkal Unni, Prathyush P. Menon, Lorenzo Livi, Mark R. Wilson, William R. Young, Helen M. Bronte-Stewart, Krasimira Tsaneva-Atanasova

**Affiliations:** ^1^Department of Mathematics, College of Engineering Mathematics and Physical Sciences, University of Exeter, Exeter, United Kingdom; ^2^Department of Computer Science, College of Engineering Mathematics and Physical Sciences, University of Exeter, Exeter, United Kingdom; ^3^Departments of Computer Science and Mathematics, University of Manitoba, Winnipeg, MB, Canada; ^4^Sport & Health Sciences, University of Exeter, Exeter, United Kingdom; ^5^Department of Neurology and Neurological Sciences, Stanford University School of Medicine, Stanford, CA, United States; ^6^Department of Bioinformatics and Mathematical Modeling, Institute of Biophysics and Biomedical Engineering, Bulgarian Academy of Sciences, Sofia, Bulgaria; ^7^Living Systems Institute, University of Exeter, Exeter, United Kingdom

**Keywords:** Parkinson's disease, freezing of gait, neural networks, naive bayes, random forrest

## Abstract

Freezing of gait (FoG) is typically a symptom of advanced Parkinson's disease (PD) that negatively influences the quality of life and is often resistant to pharmacological interventions. Novel treatment options that make use of auditory or sensory cues might be optimized by prediction of freezing events. These predictions might help to trigger external sensory cues—shown to improve walking performance—when behavior is changed in a manner indicative of an impending freeze (i.e., when the user needs it the most), rather than delivering cue information continuously. A data-driven approach is proposed for predicting freezing events using Random Forrest (RF), Neural Network (NN), and Naive Bayes (NB) classifiers. Vertical forces, sampled at 100 Hz from a force platform were collected from 9 PD subjects as they stepped in place until they at least had one freezing episode or for 90 s. The F1 scores of RF/NN/NB algorithms were computed for different IL (input to the machine learning algorithm), and GL (how early the freezing event is predicted). A significant negative correlation between the F1 scores and GL, highlighting the difficulty of early detection is found. The IL that maximized the F1 score is approximately equal to 1.13 s. This indicates that the physiological (and therefore neurological) changes leading to freezing take effect at-least one step before the freezing incident. Our algorithm has the potential to support the development of devices to detect and then potentially prevent freezing events in people with Parkinson's which might occur if left uncorrected.

## 1. Introduction

Parkinson's disease (PD) is a neurodegenerative disorder affecting more than 16 million people worldwide (1). The etiology of the disorder involves the death of dopaminergic neurons in the substantia nigra pars compacta of the basal ganglia (2). Both cognitive (e.g., depression and sleep difficulties) and motor (e.g., tremor, rigidity, bradykinesia, changes in speech, and FoG) symptoms are associated with PD (3).

The onset of FoG typically occurs in advanced stages of the disease progression (4) and is one of the most debilitating features of PD, affecting the well-being and quality of life of between 20 and 80% of patients. Additionally, falls and FoG are interconnected in PD patients (5, 6). Anxiety often experienced during freezing episodes hinders movement automaticity and efficiency, making a fall more likely when a step is finally initiated (6). Though no permanent cure is available, current therapies include medications (e.g., Levo-Dopa) and Deep Brain Stimulation (DBS) (7–11). However, freezing pathology is often resistant to pharmacological and surgical interventions, thus emphasizing a need to develop alternative strategies to help people avoid freezing in daily life (12).

Although some recent studies on detecting and predicting freezing from wearable sensors have been published [e.g., electrocardiography, skin conductance, and accelerometry; (1, 13)], there is also a need for further work exploring other signals. Several cueing strategies (e.g., auditory and visual) exist that often induce clear benefits in terms of stabilizing gait (12, 14, 15) and, potentially delaying FoG onset (7). However, it is not practical for users to continuously listen to sensory cues and focus their attention on walking as sensory systems such as vision are required for other functions such as route planning and avoiding hazards. Therefore, a more efficient strategy would be to initiate sensory cues at a time when the user needs them most (i.e., at a time when they are about to freeze). Understanding behavioral factors that are predictive of upcoming freeze events could help to progress our understanding of underlying neurophysiological mechanisms that cause and/or exacerbate freezing.

While there are several recent contributions applying machine learning to Parkinsonian tremors (16) and gait analysis (17) as well as the freezing of gait detection (13, 18), to our knowledge, no work has used measurements of stepping actions, using kinetic data alone to predict upcoming freezing events. Therefore, this study aims to forecast freezing events from the kinetic stepping data.

Specifically, this work makes the following contributions:

- The proposed approach accurately and robustly predicts freezing events from force data obtained while stepping. This is accomplished using machine learning techniques—random forest, neural networks and nave bayes, and the results are compared. “Kinetic” data, as is used here, likely contains subtleties in the loading and unloading phases of the stance that would directly translate to clear kinematic outcome measures. In the proposed approach we do not translate the kinetic data into kinematic data such as position and velocity.
- Systematic analysis demonstrating how the performance changes with respect to different windowing parameters that characterize the training data.

The remainder of the paper is structured as follows. The methodology section 2 starts with the preprocessing and labeling of the data. Results are discussed in section 3. Conclusions and future research directions in section 4.

## 2. Methodology

**Figure 1:**
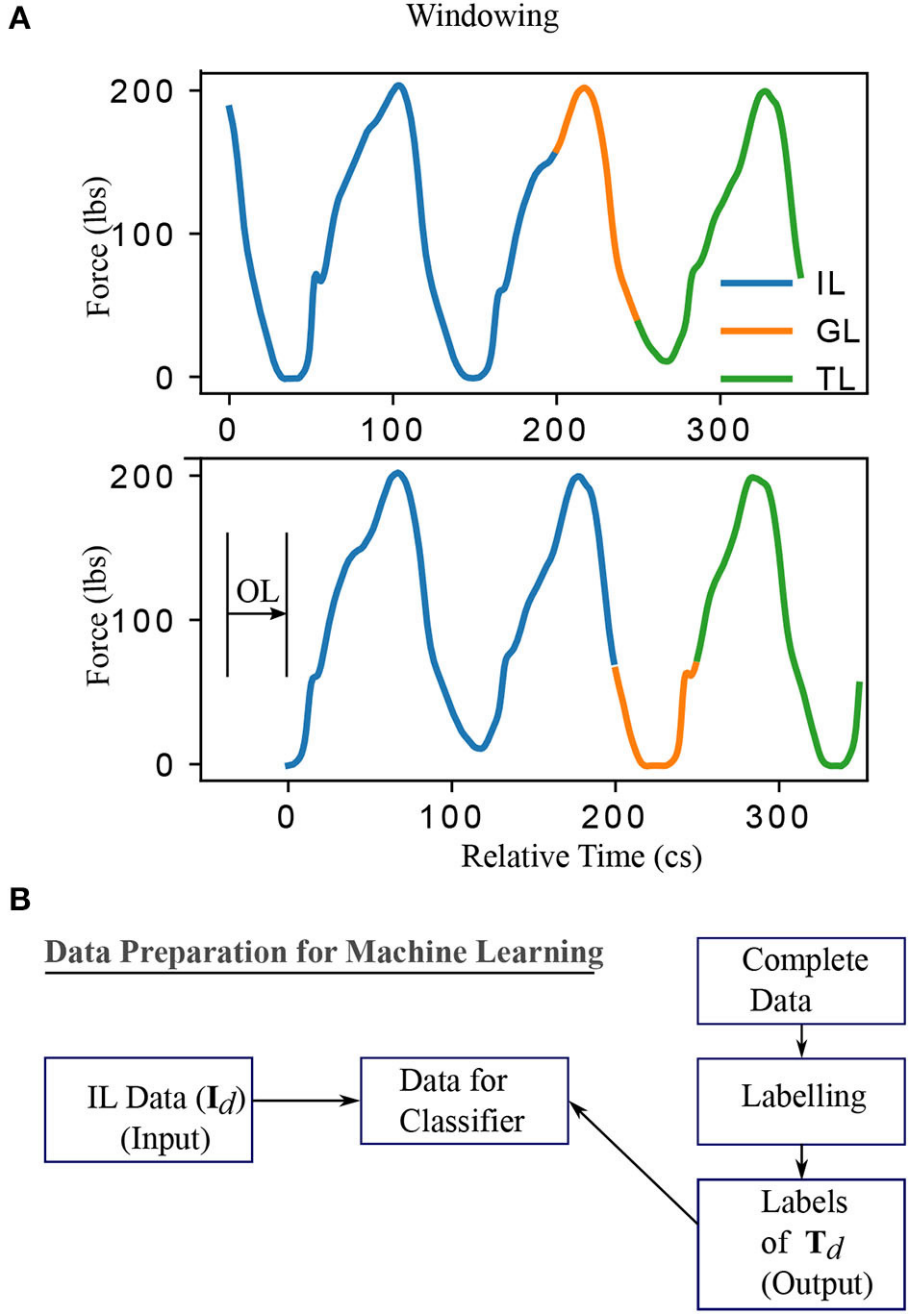
The windowing and data conditioning methodology. **(A)** The data for machine learning are obtained by considering a specific instance of input, gap, target, and offset length. By varying these parameters, one obtains different datasets and, accordingly, different classification problems. Specifically, IL (Input Length) and label extracted from TL (Target Length) determine the input and output, defining the classification problem. GL (Gap Length) indicates how early the event can be detected. OL (Offset Length) determines the distance between two windows. A larger value of TL is shown for clarity while it is chosen to be 1 in this work to predict the immediate event. **(B)** Summary of data preparation for training/testing the classifiers. IL and the labels obtained from TL region of the data formed the input and output data respectively. The labeling procedure is given in Supplementary Section 2. This procedure of data conditioning is repeated for all data obtained by moving the window at an offset of OL. Centiseconds (cs) is adopted in this work as the unit of time as the sampling frequency is 100 Hz. This results in the discrete number of time points available for analysis in any time interval matching exactly the quantity of time elapsed from start to end of that interval.

The methodology constitutes of preprocessing the data, generation of models from this data using machine learning approaches and testing the obtained models to compute performance scores. The input-output pairs of training and testing data are generated by windowing the data as shown in Figure 1A. The data vector contained in the IL (Input Length) is represented as **I**_*d*_ and the dimension of the data vector depend on the value of IL and the sampling frequency. Similarly, vectors **T**_*d*_ and **G**_*d*_ depend on TL (Target Length) and GL (Gap Length), respectively. IL, GL, OL (Offset Length), and TL are windowing parameters for data preprocessing and not internal parameters of the classifier. The label for **I**_*d*_ is assigned as “one that is representative of a future freezing” (labeled “1”) or “not freezing” (labeled “0”) episode using a binary value that corresponds to the **T**_*d*_ using an automated process described in labeling section. This results in **I**_*d*_ and **T**_*d*_ forming the input and output for training the classifiers as illustrated in Figure 1B. In summary, as shown in Figure 1A the sample window is split into three sections IL, GL, and TL. The force data in the first part of the window (IL) is used as the input to forecast the freezing state in TL part of the window (“1” for freezing and “0” for not freezing). GL is the temporal distance between IL and TL and it determines how early the predictions can be made, while, OL determines the offset interval between windows. When the total window length (IL+GL+TL) is greater than OL there will be overlap between windows. The details about windowing are given in the preprocessing section. The data used for training/testing depends on windowing parameters, therefore the impact of these parameters on classification performance is determined. The following subsections provide the details of preprocessing and training/testing of machine learning algorithms using the data.

Data used in this work constitute data from a previous study (19), collected using ‘two force plateslates customized to fit a SMART Equitest from Neurocom’ (at 100 Hz until they at least had one freezing episode or for a duration of 90 s). Out of 19 patients recruited, data from 9 patients with freezing episodes are used in this work. The details of the data such as experimental protocol, inclusion and exclusion criteria, patient background details are provided in the Supplementary Section 1. The methodology used for labeling is also provided in Supplementary Section 2.

### 2.1. Preprocessing

A PCA (principal component analysis) of the data in a 3D-coordinate system measuring force and moments in x (frontal), y (lateral), and z (vertical) directions was performed and it showed the largest variance in the vertical direction. Hence, only the vertical (z) coordinate is used here for the analysis. A graphical representation of the data preprocessing procedure is shown in Figure 1A. The data has been windowed into IL, GL, and TL. The label for IL is assigned by extracting the corresponding label of the TL part of the data as indicated in Figure 1B. This way the forecasting problem becomes a classification problem. The data contained in IL and the corresponding label form a training data sample. TL has been chosen to be 1 in this work to ensure the prediction of the status of the patient immediately after GL. But the procedure mentioned is general enough to include a larger TL and make predictions considering all events happening in that TL time frame. However, the procedure described is general enough for any value of TL. Window length (WL) is the total length of the data used to generate a single sample, that is the sum of IL, GL, and TL. The window of length WL is slid across the data (independently for training and testing data which is described in the cross-validation section Supplementary Section 3) with an offset (see Figure 1A) of OL to prepare multiple data samples for training and testing. This is done at the individual patient level so that cross-validation can be carried out on an individual basis. The mean and standard deviation of the quarter of cycle length of all the patients is 28.4 and 7.6 cs, respectively. Hence, the OL is chosen to be 28 cs (approximately a quarter of an average cycle length) for training and testing for all the patients. But this does not prevent one from using the tested classifier at a lower OL value to produce a higher temporal resolution in real-time prediction scenario. The OL is chosen to be well below the cycle length to avoid stepping cycles getting missed while window length WL is slid with an offset OL.

### 2.2. Classifiers and Related Parameters

The following sections describe the classifiers and the reason for choosing NB as the benchmark.

#### 2.2.1. NB as Benchmark

NB classifier acts as the benchmark classifier for this study because of the following reasons. The classifier assumes conditional independence and is very fast for supervised learning (20). That is, the predicted label ỹ is computed as $ỹ=P\left(y\right)\underset{y}{\arg\max}\prod_{i=1}^{n}P\left(x_{i}|y\right)$ where *P*(*x*_*i*_|*y*) (conditional probability of a feature *x*_*i*_ given label *y*) is computed by assuming a Gaussian distribution, where, the parameters are estimated using maximum likelihood estimation algorithm (MLE) (21). The proportionality constant *P*(*y*) is the relative frequency of the label *y*.

#### 2.2.2. NN and RF Classifiers

NN classifier can learn a non-linear function approximation and generate an appropriate decision boundary for the problem. 5 hidden layers of 100 neurons were used for supervised learning. The solver used in NN is limited memory Broyden-Fletcher-Goldfarb–Shanno (LBFGS) for faster convergence (22–24). A lower number of layers deteriorated the performance while a higher number didn't improve the results. An RF classifier is an estimator that fits a set of decision tree classifiers on sub-samples of the dataset and uses its average for prediction. Maximum depth of the tree used is 200, with a class weight of 100 for the true cases and 1 for false giving more weight to freezing prediction. The freezing phenomenon is episodic and there is a lower number of freezing episodes when compared to the number of walking cycles in PD (25). A higher class weight in RF is chosen for the true cases to address this imbalance and to signify the relative importance of freezing prediction when compared to predicting the normal stepping. To understand it's impact weights assigned to the true cases are varied from 1 to 100 with a spacing of 10 at IL = 113 and GL = 0. The RF classifier has a lesser chance of over-fitting as it uses multiple trees and averaging.

### 2.3. Classifier Comparison

The performance of three well-established classifiers RF, NN, and NB (23) is compared. The NB classifier acted as a baseline for comparison. F1-scores can be calculated in multiple ways (26). The averaged F1-score version described in (26) is used as the “F1-score” in this work. Median F1-Score across different patients is used as the performance measure for a classifier. The effect of the IL and GL on the performance measure is determined to understand the trends and optimal IL. The F1-scores claimed are after the application of “minority-vote,” while methodologies such as “majority-vote” and “minority-vote” are equally valid with their pros and cons. A short comparison of the “minority-vote” technique with the “majority-vote” methodology is also provided for a subset of parameter values for all the subjects combined. The detailed description of the parameters used for comparison of classifiers is provided in the Supplementary Section 4.

### 2.4. Procedure for Real Time Prediction

This section presents the proposed methodology for real time prediction. This is not to evaluate the performance of the algorithms. A real-time prediction scenario is illustrated for a single patient.

Moreover, to show the ability of the algorithm to control sensitivity/specificity by changing only the way of combining the models, the false positive and negative ratios are provided here for a particular patient using minority-vote and majority-vote methodology. To this end, mean false positives (MFP) and mean false negatives (MFN) of all classifiers (NN, NB, RF) combined is estimated for a majority-vote and minority-vote cases for the case shown here and their ratios are provided. False-positive and false negative rates are also computed for all the patients for comparative purposes.

In the case demonstrated, an IL = 226 cs is used and the data is supplied to the classifier by sliding the window over time. This sliding window with an OL = 10 cs replicates, the real-time prediction scenario where prediction is made after every 10 cs. The classifier has to predict the immediate possibility of a freeze giving “1” in the case of a freeze and “0” otherwise. The output of the classifier is obtained over time and shown along with ground truth (GT). The label of TL in each sample form the ground truth for that sample. An ideal classifier matches GT. No prediction is possible until the first set of data points for the time defined by IL is available. Therefore, this doesn't form part of the “union of TL data.” After this point, there is a prediction that corresponds to every TL data.

## 3. Results

The fraction of freezing episodes is approximately 34% of the overall data, the total number of freezing episodes is 174, the average duration freezing episodes is approximately 12.08 s (SD = 13.5 s) and the average number of freezing episodes per subject is 3 (SD = 1.6) per trial.

In this section we present the following results:

- Analysis of the performance of classifiers by varying the windowing parameters (IL and GL), the results of which are discussed in section 3.1.
- A demonstration of the on-line prediction is discussed in section 3.2.

### 3.1. Classification Performance

**Figure 2:**
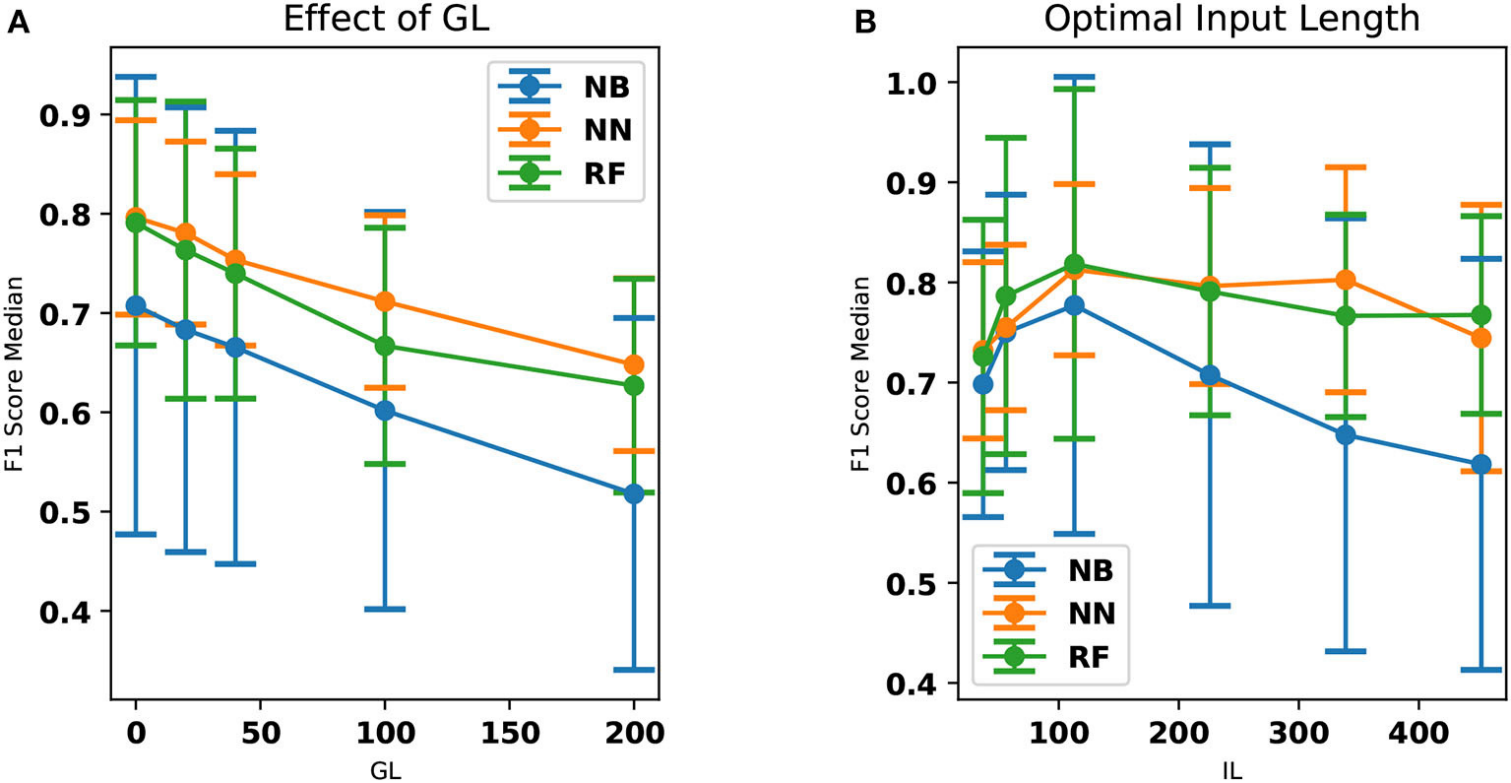
The effect of GL and IL on the median F1-scores. The standard deviation has also been provided as error bars. **(A)** The median F1-scores of NN, RF, and NB classifiers are compared here. NN classifier outperforms all other classifiers in this aspect. The performance of the classifier decreases with respect to an increase in GL. Dots and the lines drawn indicate the computed data points and the linear interpolation between them, respectively. **(B)** F1 score (median over all the patients) is shown as a function of the IL. The accuracy is shown to be optimal at an input length of 113 cs. This is particularly evident in the case of the NB and RF classifiers. Dots and the lines drawn indicate the computed data points and the linear interpolation between them, respectively.

**Figure 3:**
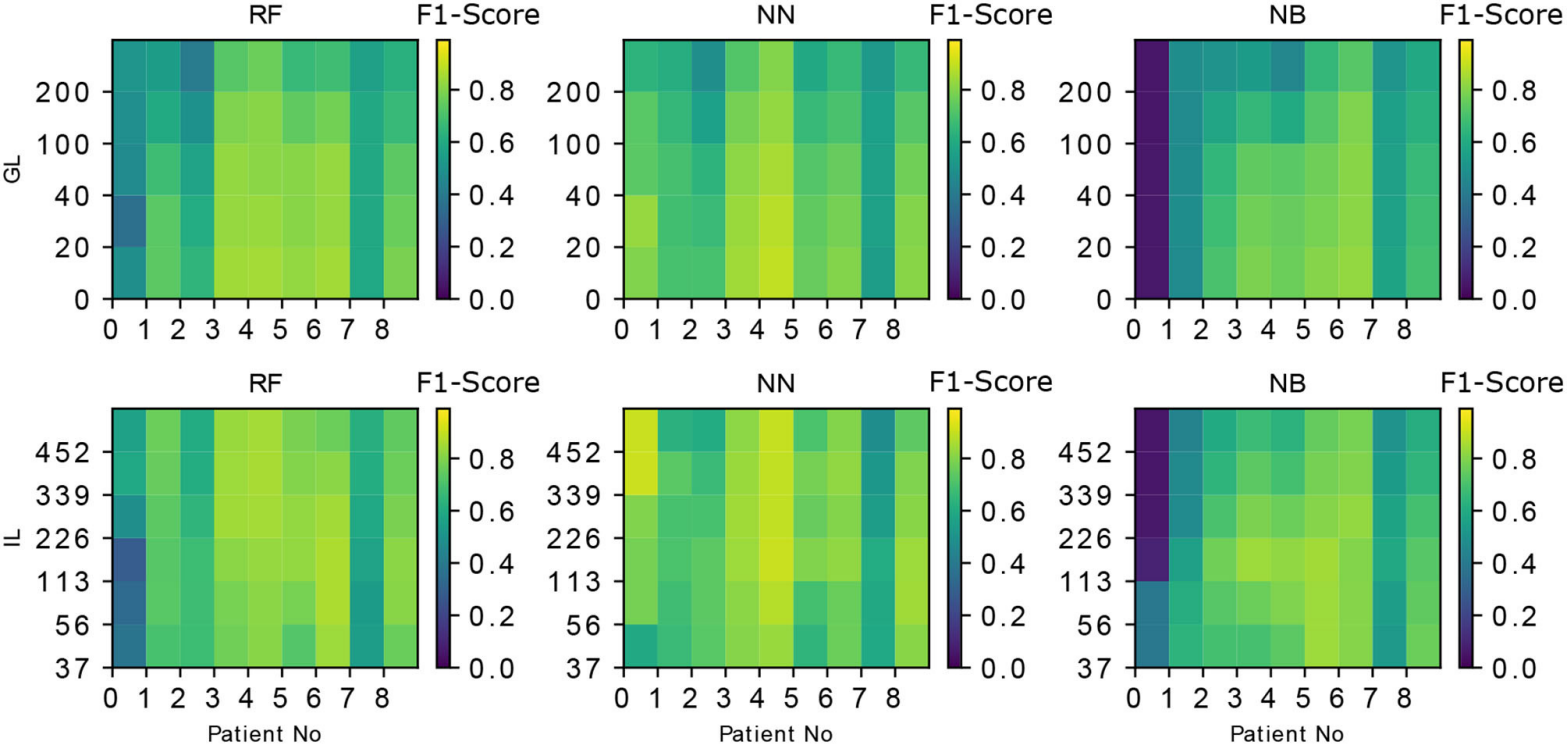
The the effect of IL, GL on the F1-scores of the classifiers is demonstrated for every classifier and every patient. IL = 226 cs is chosen when GL is varied and GL = 0 is chosen when IL is varied. The patient-specific score variation can also be noted in every case (e.g., The accuracy of the first patient (patient No. “0”) is lower for both RF and NB but NN performs better in that case). The metric used in the color-bar for comparison is the F1-score.

The performance of NN and RF is found to be superior compared to NB (Kruskal Wallis H test, *p* < 0.05 for both). This could be due to a more complex relationship between the features learned by NN and RF when compared to NB classifier. The performance of NN and RF were not statistically different (Kruskal Wallis H test, *p* ≊ 0.276). GL and the classifier performances are inversely correlated with the Spearman rank-order correlation coefficient of ≊ −0.99 and *p* < 0.01. The best input lengths for NB, RF, and NN were found to be 113, 113, and 226 cs, respectively. The neural network performs well for a range of IL (113–339 cs) while the other classifiers deteriorate the performance beyond an input length of 113 cs. Figure 3 shows the effect of IL and GL on the F1-scores of the classifiers for every patient. A constant value of IL (= 226 cs) is chosen when GL is varied and GL = 0 cs is chosen when IL is varied. The scores are found to vary between patients. While Figures 2A,B show an overall trend, there are individual differences between patients (see Figure 3), which is typical in freezing studies (1). For example, for NB classifier, patient no. 0 revealed very low scores while patient no. 5 revealed a higher score. Also, NN performs better for patient no. 4 than RF and NB. For patient no. 6 RF performs better than the other two. There are therefore individual differences in the optimal IL estimates. This points to a need for personalization and a larger data collection exercise to determine the individual patient's optimal parameter sets, categorizing participants according to the emerging freezing “sub-types” (27).

For the RF classifier, the models corresponding to different class weights tested are used to obtain the F1-scores corresponding to every patient. The median of the standard deviations of the F1-scores (across models of varying class weights) is found to be 0.03. The corresponding median of the F1-scores (averaged across models of varying class weights) is found to be 0.82. Low standard deviation indicates the results presented are robust against the class weights used as the mean/median F1-scores are more than ten times higher than the standard deviations obtained. But, one could also argue that there is a room for personalization, by making use of the individual differences which causes the dependence of the F1-scores on the weights.

### 3.2. Real-Time Prediction Demonstration

**Figure 4:**
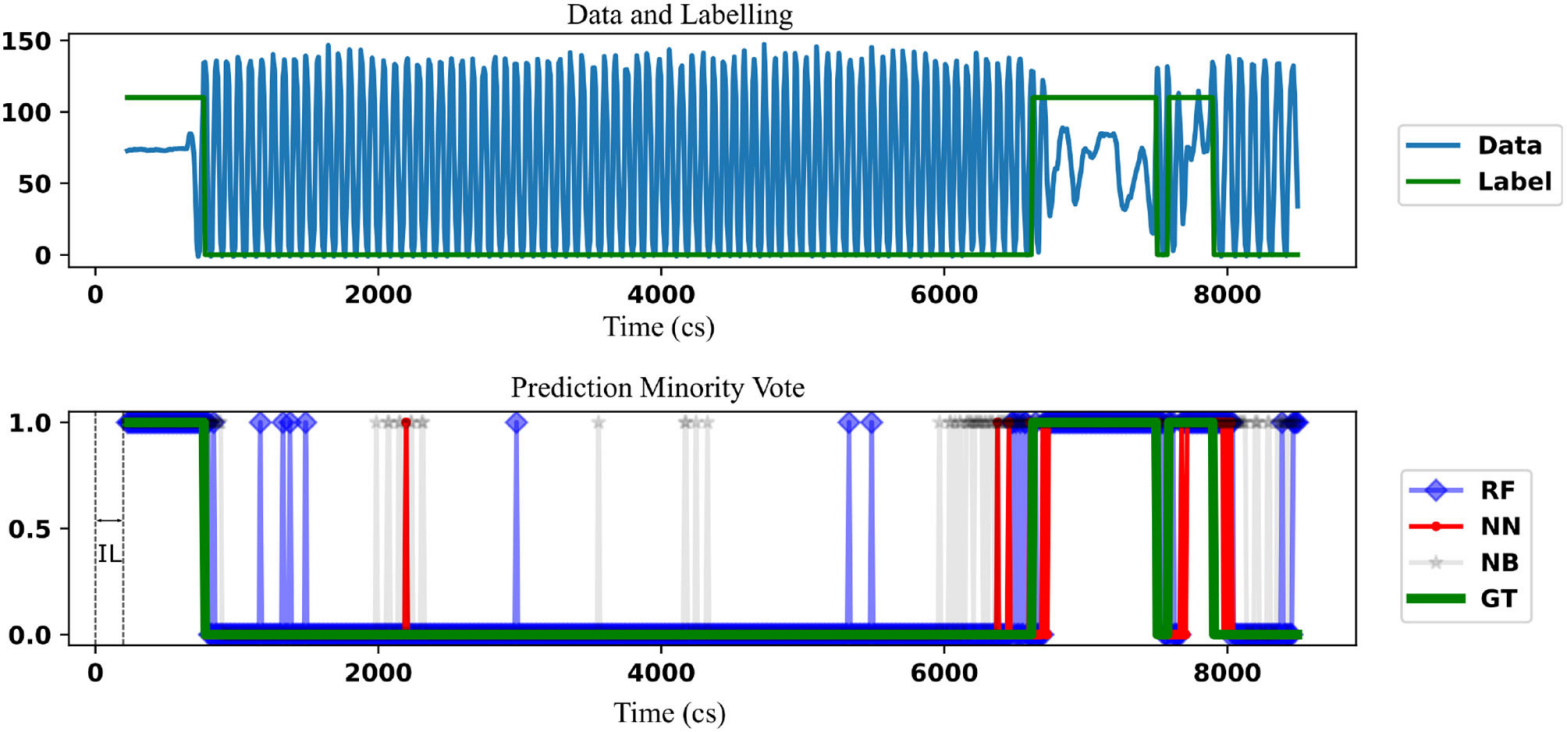
The comparison of the freezing prediction using NN, RF with the benchmark NB (using a lighter shade) is demonstrated. Data (Force in lbs) and the corresponding label for the time frame where the prediction is carried out is shown in the top figure. This is the union of the TL part of the data and corresponding label for a single patient. Label “110” is used instead of “1” to indicate freezing for clarity, that is, to keep the label well above the center of the plot. The initial IL data can't form target data as there is not enough information for prediction before that. The bottom figure indicates the comparison of the classifiers against the ground truth (GT). The prediction is started only after a time defined by IL. Parameters used are IL = 226 cs and GL = 0 cs, OL = 10 cs. In this case, NN shows fewer false positives than RF while both classifiers have less false positives than NB.

A single patient data is taken and the prediction by minority-vote is shown in Figure 4 as an example. A model which hasn't previously seen this patient's data has been used for the purpose. The example is chosen not to indicate the performance of the classifiers but to show the ability to predict in real-time (once in every 10 cs). The prediction of different classifiers is indicated in different colors. A similar result is obtained at OL = 28 cs. OL = 10 cs is demonstrated to show the ability of the classifier to be used at a higher temporal resolution than it is trained at. The NN prediction shows a lower number of false positives [a freeze (“1”) even when there is no freeze] than RF. RF classifier shows a lower number of false positives than NB. minority-vote method has lesser MFN than majority-vote method with their ratio being 0.36 : 1 (MFN for minority-vote method : MFN for majority-vote method). minority-vote method has a higher MFP than majority-vote with the ratio being 1 : 0.3 (MFP for minority-vote method : MFP for majority method). False positive and negative rates for all patients (averaged over all classifiers in minority-vote case, IL = 113, GL = 0) is found to be 0.26 (SD = 0.25), 0.20 (SD = 0.16), respectively. False-positive and negative rates for all patients (averaged over all classifiers in majority-vote case, IL = 113, GL = 0) is found to be 0.16 (SD = 0.22), 0.29 (SD = 0.18), respectively.

## 4. Discussion

Our analysis demonstrated that it is possible to predict freezing events using vertical force data from stepping. Prediction of freezing events from stepping data is addressed as a classification problem. In doing so, the data is windowed using IL, GL, TL, and OL as relevant parameters and generated the input data and output labels accordingly. Furthermore, the impact of IL and GL on the F1 scores has also been studied. A comparison of the results of the classifiers with the NB classifier was done to indicate the possibility of a complex connection between the features used for prediction.

F1-scores (> 0.8) obtained for the classification is found to be decreasing for an increased GL and this score is subject dependent. This indicates that the earlier one tried to predict a freezing event, the less accurate the prediction became. The average natural time-period of the signals is found to be approximately 113 cs in this work. Optimal performance of the classifiers in this range is indicative of the fact that, in the case of this stepping task, the physiological changes preceding a freeze take effect approximately one step before the freezing event. Despite including 174 freezing episodes, the modeling of freezes from relatively few participants is a limitation of the study and future work aims to increase the sample size to improve the generalizability.

Prediction and detection are different questions concerning freezing of gait time series data. There are several attempts to detect freezing onset. The recent study by Aich et al. (28) used accelerometer data where the authors show a detection accuracy of 88%. Whereas, in our work, we aim at prediction rather than detection of the freezing. The prediction accuracy depends heavily on the degradation of stepping (and its associated data) prior to freezing which is patient dependent (29). This dependence is also very evident in our study from the Figure 3 where the patient “0” has lower F1-scores compared to other patients for RF and NB classifiers. This highlights the necessity for a flexible, personalizable, algorithm to meet patient needs. Mazilu et al. (29), have used accelerometer data and produced an F1 score of 0.56 for prediction. Mazilu et al. (1) later show an accuracy of 71.3% using ECG and skin conductance. These studies can't be directly compared to our study because of the following reasons (1) There are limitations with the step in place task as the spatial characteristics such as “sequence effect” (30) can not be expressed in the current task. (2) The kinetic interactions between the feet and support surface are largely responsible for the kinematic changes recorded and analyzed in the previous work described above, particularly step length. As such, we argue that an evaluation of kinetics (i.e., the dynamic loading and unloading of each limb) is likely to yield more accurate predictions of resultant kinetics. (3) Also, as this work aims at real time prediction, a classification based on FoG/Pre-FoG states (29) may not be meaningful as the stream of data (in the moving window) may contain a combination of these states. Moreover, in our work, training and testing the data from different attentional focus produces a model which can work more reliably in a real life scenario where the patients are not restricted to one type of attentional focus.

In this work, the “minority-vote” methodology is used for combining the output of the classifiers. Improving safety by way of minority-vote would result in higher false positives resulting in unnecessary cues being produced and hence diminished effectiveness. But false positives resulting in triggering a cue unnecessarily is not detrimental to the patient's life but a false negative result while crossing the road can be. The fear of falling leads to immobilization of the patient and further complications such as osteoporosis, constipation, reduced fitness, social isolation etc. (6). Therefore, one could assume the benefit of avoiding a freeze far outweighs the risk of fatigue. Also, once trained the model ensembles can be combined in different ways to suit the needs of the patient. This trade-off forms one of the key limitations of this work.

Ability of the algorithm for real-time prediction and algorithm's flexibility in combining different models to enable further personalization has been demonstrated. This flexibility becomes relevant in practical cases as one would have to aim to adjust the sensitivity and specificity according to the patient needs. As the algorithm developed here generates multiple models, one could personalize this according to the patient requirements by changing the way it is combined. The majority-vote and minority-vote based methodologies demonstrated in this work form two ends of the spectrum of possible ways of combining the model outcomes. Higher false positives of minority-vote methodology is justified by the reduction in false negatives as the application necessitates higher safety.

Spatial characteristics of gait such as “sequence effect” (30) cannot be observed by force data while stepping in place. Therefore, future endeavors could aim to evaluate predictions using gait data collected during forward-walking. The methods developed here (e.g., windowing, cross-validation procedure etc.) are also suitable for accelerometer data obtained from smartphones and other wearable devices. The proposed method could be extended to other signal features and parametrized cost functionals (31) to potentially improve the prediction. Time series prediction, a multilabel classification, and personalization are future work.

In conclusion, the proposed method operating in conjunction with a sensory/visual/auditory cue (in a wearable device), could potentially be used to help a PD patient walking more efficiently with less occurrence of FoG. It could be difficult to acquire force plate data in daily life settings. However, the rapid evolution of low-cost portable devices, such as wireless force-sensing insoles provides a feasible solution to acquiring kinetic data (at least sufficient to calculate the proportion of body weight on each limb, as described here) in real time. However, the conclusion implicitly assumes providing a cue 1–2 s prior to a freeze is sufficient to address freezing, which we believe will be sufficient to provide cues such as (32). More studies have to be performed to understand “how early” and “what kind” of cue needs to be provided to reduce the chance of freezing.

## Data Availability Statement

The raw data supporting the conclusions of this article will be made available by the authors, without undue reservation.

## Ethics Statement

The studies involving human participants were reviewed and approved by the research ethics committee at Stanford University Medical School. The patients/participants provided their written informed consent to participate in this study.

## Author Contributions

MP, PM, LL, and KT-A have contributed to developing the methodology, analysis of the result, and editing the manuscript. MW, WY, and HB-S have contributed in analysis of the results and manuscript editing. All authors contributed to the article and approved the submitted version.

## Conflict of Interest

The authors declare that the research was conducted in the absence of any commercial or financial relationships that could be construed as a potential conflict of interest.

## Figures and Tables

**Table:** 

cs	Centisecond
DBS	Deep brain stimulation
FoG	Freezing of gait
GL	Gap Length
IL	Input length
MFP	Mean false positives
MFN	Mean false negatives
NB	Naive Bayes
NN	Neural Network
OL	Offset Length
PCA	Principal component analysis
PD	Parkinson's disease
RF	Random forest
SD	Standard Deviation
TL	Target Length
TS	Time series
WL	Window length

## References

[1] Mazilu S Calatroni A Gazit E Mirelman A Hausdorff JM Tröster G. Prediction of freezing of gait in Parkinson's from physiological wearables: an exploratory study. IEEE J Biomed Health Inform. (2015) 19:1843–54. 10.1109/JBHI.2015.2465134 26259206

[2] Kandel ER Schwartz JH Jessell TM Siegelbaum SA Hudspeth AJ. Principles of Neural Science. Vol. 4. New York, NY: McGraw-hill (2000).

[3] Schapira AH Chaudhuri KR Jenner P. Non-motor features of Parkinson disease. Nat Rev Neurosci. (2017) 18:435. 10.1038/nrn.2017.62 28592904

[4] Giladi N McDermott M Fahn S Przedborski S Jankovic J Stern M. Freezing of gait in PD Prospective assessment in the DATATOP cohort. Neurology. (2001) 56:1712–21. 10.1212/WNL.56.12.1712 11425939

[5] Bloem B Voermans N Aerts M Bhatia K van Engelen B Van de Warrenburg B. The wrong end of the telescope: neuromuscular mimics of movement disorders (and vice versa). Pract Neurol. (2016) 16:264–9. 10.1136/practneurol-2015-001311 26965497

[6] Bloem BR Hausdorff JM Visser JE Giladi N. Falls and freezing of gait in Parkinson's disease: a review of two interconnected, episodic phenomena. Mov Disord. (2004) 19:871–84. 10.1002/mds.20115 15300651

[7] Gilat M de Lima ALS Bloem BR Shine JM Nonnekes J Lewis SJ. Freezing of gait: promising avenues for future treatment. Parkinsonism Relat Disord. (2018) 52:7–16. 10.1016/j.parkreldis.2018.03.009 29550375

[8] Marsden C Parkes J. Success and problems of long-term levodopa therapy in Parkinson's disease. Lancet. (1977) 309:345–9. 10.1016/S0140-6736(77)91146-1 64868

[9] Breit S Schulz JB Benabid AL. Deep brain stimulation. Cell Tissue Res. (2004) 318:275–88. 10.1007/s00441-004-0936-0 15322914

[10] Anidi C O'Day JJ Anderson RW Afzal MF Syrkin-Nikolau J Velisar A. Neuromodulation targets pathological not physiological beta bursts during gait in Parkinson's disease. Neurobiol Dis. (2018) 120:107–17. 10.1016/j.nbd.2018.09.004 30196050 PMC6422345

[11] Syrkin-Nikolau J Koop MM Prieto T Anidi C Afzal MF Velisar A. Subthalamic neural entropy is a feature of freezing of gait in freely moving people with Parkinson's disease. Neurobiol Dis. (2017) 108:288–97. 10.1016/j.nbd.2017.09.002 28890315 PMC6386531

[12] Nonnekes J Snijders AH Nutt JG Deuschl G Giladi N Bloem BR. Freezing of gait: a practical approach to management. Lancet Neurol. (2015) 14:768–78. 10.1016/S1474-4422(15)00041-1 26018593

[13] Pham TT Moore ST Lewis SJG Nguyen DN Dutkiewicz E Fuglevand AJ. Freezing of gait detection in Parkinson's disease: a subject-independent detector using anomaly scores. IEEE Trans Biomed Eng. (2017) 64:2719–28. 10.1109/TBME.2017.2665438 28186875

[14] Rubinstein TC Giladi N Hausdorff JM. The power of cueing to circumvent dopamine deficits: a review of physical therapy treatment of gait disturbances in Parkinson's disease. Mov Disord. (2002) 17:1148–60. 10.1002/mds.10259 12465051

[15] Dean S Gearóid Ó Margaret R Pauline M Lois R Aimi M. Effect of auditory, visual and somatosensory cueing strategies on On-State Freezing of Gait in Parkinson's disease. Parkinsonism Relat Disord. (2020) 77:1–4. 10.1016/j.parkreldis.2020.06.010 32563079

[16] Livi L Sadeghian A Sadeghian H. Discrimination and characterization of Parkinsonian rest tremors by analyzing long-term correlations and multifractal signatures. IEEE Trans Biomed Eng. (2016) 63:2243–9. 10.1109/TBME.2016.2515760 26760968

[17] Fröhlich H Claes K De CW Van XD Michel A. A machine learning approach to automated gait analysis for the noldus catwalk (TM) system. IEEE Trans Biomed Eng. (2018). 65:1133–39. 10.1109/TBME.2017.2701204 28858780

[18] Amini A Banitsas K Young WR. Kinect4FOG: monitoring and improving mobility in people with Parkinson's using a novel system incorporating the Microsoft Kinect v2. Disabil Rehabil Assist Technol. (2019) 14:566–73. 10.1080/17483107.2018.1467975 29790385

[19] Young WR Shreve L Quinn EJ Craig C Bronte-Stewart H. Auditory cueing in Parkinson's patients with freezing of gait. What matters most: action-relevance or cue-continuity? Neuropsychologia. (2016) 87:54–62. 10.1016/j.neuropsychologia.2016.04.034 27163397

[20] Zhang H. The optimality of naive Bayes. AA. (2004) 1:3. Available online at: https://www.aaai.org/Papers/FLAIRS/2004/Flairs04-097.pdf

[21] Myung IJ. Tutorial on maximum likelihood estimation. J Math Psychol. (2003) 47:90–100. 10.1016/S0022-2496(02)00028-7

[22] Zhu C Byrd RH Lu P Nocedal J. Algorithm 778: L-BFGS-B: Fortran subroutines for large-scale bound-constrained optimization. ACM Trans Math Softw. (1997) 23:550–60. 10.1145/279232.279236

[23] Pedregosa F Varoquaux G Gramfort A Michel V Thirion B Grisel O. Scikit-learn: machine learning in Python. J Mach Learn Res. (2011) 12:2825–30. Available online at: http://www.jmlr.org/papers/volume12/pedregosa11a/pedregosa11a.pdf

[24] Liu DC Nocedal J. On the limited memory BFGS method for large scale optimization. Math Programming. (1989) 45:503–28. 10.1007/BF01589116

[25] Barthel C Mallia E Debû B Bloem BR Ferraye MU. The practicalities of assessing freezing of gait. J Parkinsons Dis. (2016) 6:667–74. 10.3233/JPD-160927 27662331 PMC5088401

[26] Opitz J Burst S. Macro F1 and Macro F1. arXiv preprint arXiv:191103347 (2019).

[27] Ehgoetz Martens KA Shine JM Walton CC Georgiades MJ Gilat M Hall JM. Evidence for subtypes of freezing of gait in Parkinson's disease. Mov Disord. (2018) 33:1174–8. 10.1002/mds.27417 30153383

[28] Aich S Pradhan PM Park J Sethi N Vathsa VSS Kim HC. A validation study of freezing of gait (FoG) detection and machine-learning-based FoG prediction using estimated gait characteristics with a wearable accelerometer. Sensors. (2018) 18:3287. 10.3390/s18103287 30274340 PMC6210779

[29] Mazilu S Calatroni A Gazit E Roggen D Hausdorff JM Tröster G. Feature learning for detection and prediction of freezing of gait in Parkinson's disease. In: International Workshop on Machine Learning and Data Mining in Pattern Recognition. ed Perner P. (Berlin; Heidelberg: Springer) (2013). p. 144–58.

[30] Chee R Murphy A Danoudis M Georgiou-Karistianis N Iansek R. Gait freezing in Parkinson's disease and the stride length sequence effect interaction. Brain. (2009) 132:2151–60. 10.1093/brain/awp053 19433440

[31] Parakkal Unni M Sinha A Chakravarty K Chatterjee D Das A. Neuro-mechanical cost functionals governing motor control for early screening of motor disorders. Front Bioeng Biotechnol. (2017) 5:78. 10.3389/fbioe.2017.00078 29326926 PMC5733372

[32] Barthel C Nonnekes J Van Helvert M Haan R Janssen A Delval A. The laser shoes: a new ambulatory device to alleviate freezing of gait in Parkinson disease. Neurology. (2018) 90:e164–71. 10.1212/WNL.0000000000004795 29263221

