# Supplementary material for: Data-Driven Prediction of Freezing of Gait Events From Stepping Data
Source: Front Med Technol. 2020 Nov 20;2:581264. doi: 10.3389/fmedt.2020.581264 (PMC8757792; doi:10.3389/fmedt.2020.581264)
Supplement: Supplementary file 1 [file Data_Sheet_1.pdf]

Supplementary Material

1 EXPERIMENTAL DATA USED

The current data constitute data from a previous study Young et al. (2016), collected using 'two force plateslates customized to fit a SMART Equitest from Neurocom'. Nineteen subjects with Parkinson's disease were recruited from the Stanford Movement Disorder clinic. The exclusion criteria were dementia, significant hearing loss, or any musculoskeletal or neurological issue (other than Parkinson's) that significantly affected their walking. Only 9 patients with freezing episodes are used in this work. All the trials with different attentional focus with freezing episodes has been used. This provides the algorithm an opportunity to train against different plausible stepping characteristics leading to a freeze. Inclusion criteria was that participants scored at least 3 or greater to the third item of the FoG questionnaire. Participants were ON medication (i.e. they were testing 1-2h after taking medication). No participants had undergone surgery for deep brain stimulation, although some were candidates undergoing evaluation for surgery. All participants had ideopathics Parkinson's and no other known neurological impairment. Participants were excluded from the study if they were unable to stand unsupported for 90s. The testing session comprised 6 trials of 90s and lasted approximately 20-30 minutes depending on the amount of time participants rested between trials. Data without FoG episodes are removed from this study resulting in at-least two trials per subject. The detailed experimental protocol is given in Young et al. (2016).

This Force plate data was collected at 100 Hz (uniformly sampled) from the subjects as they stepped in place until they at least had one freezing episode or for a duration of 90 s. The data included force and moments in all three coordinates as time series (TS). The participant characteristics are as follows, age was 62.827 ($\pm$ 8.82), Unified Parkinson Disease Rating Scale (UPDRS) score of 32.6 ($\pm$ 21.33), Hoehn and Yahr Scale (H & Y) of 2.4 ($\pm$ 0.53), years since diagnosis being 10.71 ($\pm$ 6.01) Young et al. (2016).

2 LABELLING

This subsection describes how the data labels are generated, which is considered as ground truth (ideal expected result) in the machine learning algorithms. The data is labelled into freezing and non-freezing using a modified version of the criteria provided in Nantel et al. (2011). Modifications to the criteria are given here. For scaling, the average of the peaks is used in this work as it is closer to the body-weight rather than the max of the peaks in contrast to Nantel et al. (2011). This is done to account for the fact that the force exerted can be slightly more or less than the actual bodyweight of the subject due to the acceleration of the subject's movement. In Nantel et al. (2011) freezing intervals are defined as *abnormally long* intervals between peaks where *abnormally long* is defined as 1.2 times the means of the periods of the previous three intervals or 2 times the mean period of the trial. In this work, *abnormally long* window length for detecting freezing is defined to be 1.5 times the average time-period of all the cycles across all the patients (mean cycle length). OL is also chosen to be lower than the *abnormally long* window length to avoid missing any freezing regions. The method which uses the previous three cycles for computing the time-period as given in Nantel et al. (2011) is not used as it is not applicable in the case where freezing happens during the initiation (or before three steps are completed) and uniform criteria are needed to be used throughout. A visual examination has been conducted as suggested in Nantel et al. (2011) to assure the validity of the method. Moreover, video recordings were used to qualify FOG events in Young et al. (2016) and, for trials analysed here there were no discrepancies between FOG events detected by the algorithm and visual inspection. Therefore, there is justification to assume that the detection algorithms used here

are, at least in the case of the current data, accurate in determining FOG events. This way a binary label for every time point in the data as freezing (‘1’) or non-freezing (‘0’) is obtained. In this study, a correct prediction of FoG is considered to be a positive outcome as far as the performance of the ML algorithm is concerned. This is applicable when one refers to terms such as false positives and false negatives.

3 CROSS VALIDATION

The training, testing, and validation is carried out in the following manner. A version of leave one out cross-validation (LOOCV) is carried out by first leaving all samples of one patient out. That is $\{1...N-1\}$ patient data samples were chosen from N (number of patient data sets available) as *‘model generation set’* and N^{th} one as the *‘unseen set’*. $\{1...N-2\}$ patient data samples were chosen from the *‘model generation set’* for training (*‘training set’*) and $(N-1)^{th}$ one as the *‘testing set’* generating a model to be tested on the *‘unseen set’*. Then another subset of the *‘model generation set’* of cardinality $N-2$ is chosen as *‘training set’* leaving the rest as *‘testing set’*. Then the process is repeated $N-1$ times generating $N-1$ models. These models were then tested on the *‘unseen set’* to generate $N-1$ performance scores. Successively, the process is repeated with another subset of the overall patient data set chosen as the *‘model generation set’* and the rest as *‘unseen set’* resulting in $N(N-1)$ performance scores, that is $N-1$ scores for each patient. F1 score Opitz and Burst (2019) is used to measure the performance and the median of $N(N-1)$ performance scores (with $N=9$) were used to compare different classifiers. An advantage of using this method is the generation of an ensemble of models which could be combined in different ways to inform the design of patient-specific interventions.

3.1 Integrating the Classifier Outcomes

As described in the cross-validation section, once an ensemble of $N-1$ models is generated, one needs to combine their outcomes. A ‘minority vote’ is used for combining the outputs of $(N-1)$ models to test the *‘unseen’* case. The minority vote is defined as a case where an ‘or’ gate is used to combine the binary classifier outputs. An ‘or’ gate produces a ‘0’ for two inputs if and only if both the inputs are zeros, every other case results in ‘1’. This methodology is used in this work to reduce the chances of false negatives as the application demands safety.

‘Majority vote’ methodology is another alternative for combining the classifier outcomes, where, an ‘output’ is selected when the majority of the classifiers produce that ‘output’. This method is also tested for a subset of the parameters for all the subjects to compare with the ‘minority vote’ technique.

4 PARAMETER ANALYSIS PROCEDURE

The methodology used for the analysis of the windowing parameters is described in this section. A set of F1-scores Haghighi et al. (2018) Opitz and Burst (2019) are generated by different combinations of patients and windowing parameters. That is, when the impact of IL is investigated, scores are generated by fixing GL (to 0 cs) and classifier type (to one of NN, NB or RF), and, varying IL and patient identification numbers. The specific discrete values of IL chosen are $[37,\ 56,\ 113,\ 226,\ 339,\ 452]$ and patient identification numbers are $[0,\ 1,\ 2,\ 3,\ 4,\ 5,\ 6,\ 7,\ 8]$. The average cycle length of the signals is approximately 113 cs forming the rationale for choosing the grid for IL. This produces a 2-dimensional array that corresponds to every patient and every IL. Median of these scores across the patient dimension is computed to generate a single performance measure for every IL. This procedure is carried out for all the classifier types to generate multiple 2-dimensional arrays. Similarly, when the impact of GL is investigated, scores are generated by fixing IL (to 226 cs) and classifier type (to one of NN, NB or RF), and, varying GL for all patients; successively computing the median across the patient dimension. GL part of the data is not given as an input to the classifier but it rather forms a parameter to determine how early one can predict the event. The

discrete grid used for GL is $[0,\ 20,\ 40,\ 100,\ 200]$ for understanding the prediction accuracies closer to and away from the freezing event. This is also extended in a similar way to all classifier types. Class weights for the true-cases were varied from 1 to 100 for the RF classifier with windowing parameters set as GL = 0 cs and IL = 113 cs. The models generated by varying class weights are tested to obtain the F1-scores corresponding to every patient. The median of the standard deviations of the F1-scores (across different models of varying class weights) and the corresponding median of the F1-scores (averaged across different models of varying class weights) are computed to understand the effect of class weights. Kruskal Wallis H test has been used for comparing the classifiers as we do not assume normality. The Spearman rank-order correlation coefficient is used for computing correlations.

REFERENCES

Haghighi, S., Jasemi, M., Hessabi, S., and Zolanvari, A. (2018). PyCM: Multiclass confusion matrix library in python. *Journal of Open Source Software* 3, 729. doi:10.21105/joss.00729

Nantel, J., de Solages, C., and Bronte-Stewart, H. (2011). Repetitive stepping in place identifies and measures freezing episodes in subjects with parkinson's disease. *Gait & posture* 34, 329–333

Opitz, J. and Burst, S. (2019). Macro f1 and macro f1. *arXiv preprint arXiv:1911.03347*

Young, W. R., Shreve, L., Quinn, E. J., Craig, C., and Bronte-Stewart, H. (2016). Auditory cueing in parkinson's patients with freezing of gait. what matters most: action-relevance or cue-continuity? *Neuropsychologia* 87, 54–62
